# Primary Biliary Cholangitis Associated with Ulcerative Colitis: Case Series and Literature Review

**DOI:** 10.3390/medicina61010044

**Published:** 2024-12-30

**Authors:** Qi Li, Ye Zong

**Affiliations:** National Clinical Research Center for Digestive Diseases, State Key Laboratory for Digestive Health, Department of Gastroenterology, Beijing Friendship Hospital, Capital Medical University, Beijing 100050, China; helenliqi_0001@163.com

**Keywords:** ulcerative colitis, primary biliary cholangitis, extraintestinal manifestations, liver biopsy

## Abstract

*Purpose*: To study the coexistence of ulcerative colitis (UC) and primary biliary cholangitis (PBC). *Methods*: The Beijing Friendship Hospital patient database was explored to identify patients presenting both UC and PBC from January 2015 to July 2024. By a review of the literature, the characteristics of UC patients who experienced PBC was summarized. *Results*: We identified 890 UC patients and reported 4 individuals who suffered from UC and PBC. Compared to the general population, UC patients seem to have a higher risk of PBC. Only 28 cases of UC with PBC have been reported so far. Most patients were diagnosed with UC prior to PBC. Once UC patients are complicated with PBC, they might experience more than one extraintestinal manifestation (EIM). Shared susceptibility genes, certain bacterial infections, and common immune-mediated mechanisms may be involved in the pathogenesis of UC patients with PBC. *Conclusions*: Although the coexistence of UC and PBC is uncommon, PBC should be considered in UC patients with hepatobiliary disorders.

## 1. Introduction

Ulcerative colitis (UC) is a type of inflammatory bowel disease (IBD). It is an autoimmune condition and characterized by continuous, diffuse, and superficial mucosal inflammation extending from the rectum to the more proximal colon [1,2]. Over a third of UC patients experience at least one extraintestinal manifestation (EIM) that involves multiple organs, such as the joints, eyes, skin, and liver [2]. Approximately 20% of UC patients present with liver disorders. These diseases, which always induce liver dysfunction, can be divided into concomitant liver diseases and hepatobiliary manifestations. The most common concomitant liver diseases in UC patients are non-alcoholic liver disease and cholelithiasis, while the most common UC-related hepatobiliary manifestation is primary sclerosing cholangitis (PSC) [3].

Primary biliary cholangitis (PBC) is a cholestatic, chronic autoimmune liver disease, which is characterized by inflammation of the small- and medium-sized bile ducts, eventually resulting in cirrhosis [4]. Extrahepatic manifestations are seen in up to 73% of PBC patients. The most common ones are Sjogren’s syndrome (3.5–73%), thyroid dysfunction (5.6–23.6%), and systemic sclerosis (1.4–12.3%) [5]. Although several cases of PBC patients with UC and a few with Crohn’s disease (CD) have been reported, the association between PBC and IBD is not widely recognized.

Evidence suggests that UC patients have a 30 times higher risk of PBC [6]. However, only 24 patients of UC with PBC have been published so far. We explored our hospital’s patient database to identify patients presenting both UC and PBC from January 2015 to July 2024. The coexistence of the two diseases was recognized in 4 individuals out of 890 UC patients. We hereby report these four cases who have suffered from both UC and PBC.

## 2. Case Presentation

### 2.1. Case 1

A 55-year-old man was diagnosed with UC in 2010 because of chronic diarrhea. Mesalamine was prescribed, and his symptoms were resolved. In June 2022, he was admitted to our hospital because of abdominal pain and diarrhea (4–6 times per day) for 2 weeks. At that time, he had been under mesalamine maintenance treatment (4 g/d) for 1 year. He had a history of PBC for 4 years, and 12.5 mg/kg/d ursodeoxycholic acid (UDCA) was taken for treatment. Chronic renal insufficiency was found 2 years prior. Moreover, he had the symptoms of dry mouth and dry throat, which made it difficult to swallow for 1 year. On admission, he had no fever, no tachycardia, no skin rashes, no joint pain, and no swelling.

**Figure 1:**
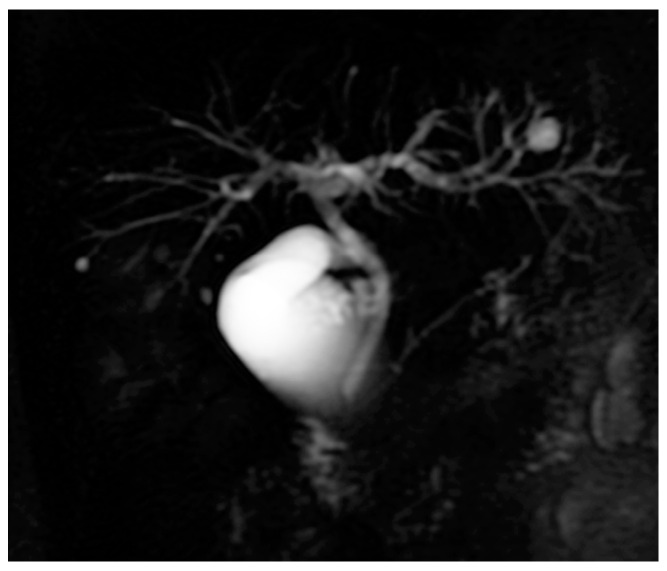
MRCP in case 1. No intra- or extrahepatic biliary abnormalities were revealed.

The total white blood cell (WBC) count and C-reactive protein were normal. However, the erythrocyte sedimentation rate (ESR) was elevated to 39 mm/1 h. The hemoglobin level was 107 g/L. The colonoscopy indicated moderately to severely active UC (pancolitis), with a Mayo Score of 3. The total bilirubin (T-BIL), direct bilirubin (D-BIL), aspartate aminotransferase (AST), and alanine aminotransferase (ALT) levels were normal. The levels of serum alkaline phosphatase (ALP), and gamma glutamyl transpeptidase (GGT) were elevated to 244 U/L and 91 U/L, respectively. The anti-nuclear antibody (ANA) was positive (1:160), as was the serum anti-M2 antibody (127.58 U/mL). ANCA were absent in the sera. Serum IgG and IgM levels were increased to 2120.0 mg/dL and 367.0 mg/dL, respectively. The results of the serum IgG subclass measurements demonstrated that elevated serum IgG levels came from IgG1, IgG2, and IgG3, not IgG4. In order to differentiate PSC, magnetic resonance cholangiopancreatography (MRCP) was performed, and no intra- or extrahepatic biliary abnormalities were revealed (Figure 1).

**Figure 2:**
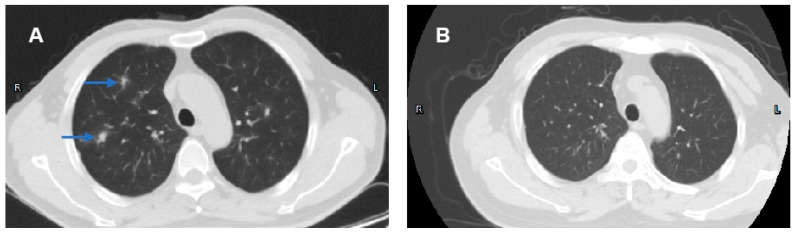
CT scan before (**A**) and after (**B**) the steroids therapy. (**A**) CT scan before the treatment (June 2022). The results displayed interstitial inflammation and multiple ground-glass nodules (blue arrows) in lungs. (**B**) CT scan after the treatment (December 2022). Interstitial inflammation and multiple ground-glass nodules decreased significantly in lungs after steroid therapy.

Ophthalmological examination, salivary gland ultrasonography, and labial salivary gland biopsy were completed. The Schirmer’s test was positive. Diffuse lesions were involved in bilateral parotid and submandibular glands. The histology of the labial salivary gland specimen showed diffuse lymphocyte infiltrations. These results strongly supported the diagnosis of Sjogren’s syndrome (SS) for this patient. The serum creatinine and urea nitrogen levels were increased to 143.8 umol/L and 8.48 mmol/L, respectively. The levels of serum albumin and albuminuria were within the normal range. Urine α1-microglobulin and N-acetyl-β-D-glucosaminidase (NAG) were increased to 25.20 mg/dL and 35.5 U/L, indicating tubulointerstitial nephritis (TIN). Interestingly, the computed tomography (CT) scan suggested the co-existence of interstitial lung disease (ILD) (Figure 2A). However, he had no symptoms related to lung disease. And no evidence of lung infection was found.

Methylprednisolone 60 mg/d was given intravenously for 1 week, followed by oral steroids (methylprednisolone 60 mg once daily, decreasing the dose every two weeks by 5 mg). Mesalamine 4 g/d was also given. As for SS, hydroxychloroquine 0.2 g twice daily was prescribed. UDCA was continuously taken for PBC treatment. After the one-week treatment, the symptoms of abdominal pain and diarrhea were resolved. After 3 months, the levels of hemoglobin and ESR returned to normal. However, the levels of ALP and GGT were not changed. Interestingly, the symptoms of dry mouth and dry throat were relieved after 3 months treatment. And urine α1-microglobulin and N-acetyl-β-D-glucosaminidase were decreased to normal levels. In addition, the repeat CT scan demonstrated decreased interstitial inflammation and reduced lung nodules with time (Figure 2B). These results indicated this patient was in recovery from TIN and ILD due to steroid therapy.

Unfortunately, UC relapsed when the methylprednisolone dose was decreased to 5 mg/d. Steroid dependence was considered since no evidence of infection was found. After that, he received treatment with 5 mg/kg infliximab. The therapy was not successful even though the infliximab dose was increased to 10 mg/kg (infliximab plasma concentration was over 15 μg/mL and anti-infliximab antibody tests were negative). In September 2023, the treatment was switched to ustekinumab, which proved to be effective. Until June 2024, the patient was still in remission.

### 2.2. Case 2

A 44-year-old woman was initially diagnosed with UC (pancolitis) at the age of 33 in 2013 when complaining of chronic abdomen pain and diarrhea. As treatment, 5-ASA (mesalamine 4 g/d) was taken, and her symptoms were resolved. Then, mesalamine (4 g/d) was continued for maintenance therapy. Thereafter, she had recurrent episodes of bloody diarrhea, which could be relieved with the addition of oral steroids to her treatment.

**Figure 3:**
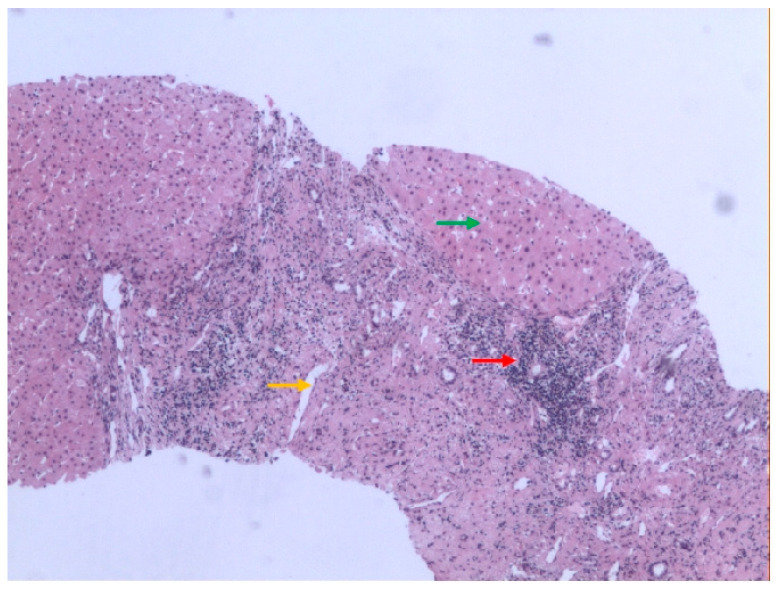
Liver biopsy specimen in case 2. The result of pathology showed mild swelling of hepatocytes (green arrow), lymphocyte infiltration in the portal area (red arrow), small bile duct proliferation (yellow arrow), and pseudolobule formation. Hematoxylin and Eosin (H&E) staining, 10 × 10.

An increase in AST level (about 300 U/L) was found at the same time she was first diagnosed with UC in 2013. UDCA was prescribed and the level of AST fluctuated between 60 and 250 U/L. In January 2016, she was admitted to our hospital for further diagnosis. The total WBC count was normal, while the hemoglobin level was decreased to 10.3 g/dL. The results of the platelet count and prothrombin time (PT) were within the normal range. T-BIL and D-BIL numbers were normal; however, the levels of ALT (70 U/L), AST (64 U/L), ALP (250 U/L), and GGT 59 U/L were all elevated. The ANA titer was 1:320, and other antibody tests, such as for AMA-M2, SP100, and GP210, were all negative. The level of IgG was increased to 2240.0 mg/dl; however, the levels of IgM and IgA were all normal. MRCP showed liver cirrhosis and splenomegaly, but no intra- or extrahepatic biliary abnormalities were revealed. Percutaneous liver biopsy showed mild swelling of hepatocytes, lymphocyte infiltration in the portal area, small bile duct proliferation, and pseudolobule formation (Figure 3). This result indicated PBC with cirrhosis.

Unfortunately, liver cirrhosis progressed even though UDCA was continued. By the end of 2018, massive gastrointestinal bleeding occurred twice in one month. After fluid resuscitation and blood transfusion, endoscopy was performed. Esophageal varices were detected, and endoscopic variceal ligation (EVL) was completed. Worsening the situation, the levels of T-BIL (526.41 umol/L), D-BIL (363.15 umol/L), and ALT (85 U/L), AST (162 U/L) were all significantly increased. PTA greatly decreased to 30.2%. Considering the situation of liver failure, liver transplantation was suggested. Based on the fact that UC was in remission (colonoscopy demonstrated mucosal healing in the colon and rectum with a Mayo endoscopic subscore of 1), the surgery was finally completed in 2019. Thereafter, she was followed up regularly at the clinic. Methylprednisolone (8 mg once per day), tacrolimus (2.5 mg twice per day), and mycophenolate mofetil (750 mg twice per day) were prescribed for long-term anti-rejection therapy. UDCA and mesalamine were still continued.

### 2.3. Case 3

A 64-year-old woman was admitted to our hospital in June 2023 because of yellowing of the skin and sclera, present for one month. On admission, she had no fever, no weight loss, no abdominal pain, and no itching. The stool color was normal, but the urine color was dark. She had a history of UC (proctitis) for 9 months. Oral 5-ASA (mesalamine 4 g/d) and sulfasalazine suppositories (500 mg twice a day) were prescribed for therapy. Although the patient stopped using medications due to jaundice one month prior, she showed no signs of UC relapse.

The result of the complete blood count was normal. The level of prothrombin time (PT) was within the normal range. The levels of ALT (304 U/L), AST (145.8 U/L), ALP (240 U/L), GGT 390 U/L, T-BIL (68.39 umol/L), and D-BIL (40.21 umol/L) were all increased. The results of ANA (1:320) were positive, as were the results for AMA-M2 (144.05 U/mL), SP100 (95.34 U/mL), and GP210 (132.41 U/mL). The level of IgG was elevated to 1910.0 mg/dL; however, the level of IgM was normal. Results for hepatitis B surface antigen and anti-hepatitis C virus antibody were negative. MRCP showed liver cirrhosis, but no intra- or extrahepatic biliary abnormalities were revealed. Percutaneous liver biopsy confirmed the diagnosis of PBC (stage III-IV) and excluded concomitant non-alcoholic fatty liver disease or autoimmune liver disease. Colonoscopy described the normal mucosa in the whole colon and rectum which indicated that UC was in remission.

UDCA 12.5 mg/kg/d was prescribed. After 3 months, the symptoms of jaundice disappeared, and the levels of ALT, AST, ALP, GGT, T-BIL, and D-BIL were all decreased to normal. Until June 2024, UDCA was still continuously taken. PBC and UC were all in remission.

### 2.4. Case 4

A 49-year-old woman was initially diagnosed with “liver cirrhosis” in 2015. Esophageal varices were detected in 2016 and EVL was performed 3 times from 2016 to 2019 for gastrointestinal bleeding prevention. In 2017, partial splenic artery embolization was completed because of hypersplenism. Percutaneous liver biopsy was carried out when the last EVL was performed in 2019. The results suggested the diagnosis of overlap syndrome (PBC and PSC). From then on, UDCA was prescribed for therapy. In June 2023, the patient was admitted to our hospital complaining of abdominal distension and jaundice for 8 years and worsening over the past 2 months. On admission, she had a history of UC (pancolitis) for 2 years. Vedolizumab (VDZ) was prescribed for remission induction and maintenance therapy.

The result of the complete blood count indicated pancytopenia (WBC: 2.94 × 10^9^ /L, Hb 50 g/L, PLT: 32 × 10^9^ /L). PT increased significantly to 22 s. The levels of T-BIL (185.78 umol/L) and D-BIL (105.46 umol/L) were elevated; however, the levels of ALT, AST, ALP, and GGT were within the normal range, which demonstrated biliary enzyme separation. The ANA titer was 1:160. The results for AMA-M2 and SP100 were positive, but those for GP210 were negative. The levels of IgG and IgA were increased to 1960.0 mg/dL and 440 mg/dL; however, the level of IgM was normal. The CT scan showed liver cirrhosis, splenomegaly with partial splenic infarction, small amount of ascites, portal hypertension, collateral circulation formation, esophagogastric varices, and uneven calibers of intrahepatic bile ducts.

The serum creatinine and urea nitrogen levels were increased rapidly in 1 week, from 266.9 umol/L and 8.43 mmol/L to 374.8 umol/L and 11.27 mmol/L, respectively. The 24 h urine volume was within the normal range. Urine total protein was increased to 0.45 g (24 h), while the level of albumin was decreased to 25.2 g/L. Urine α1-microglobulin (2.99 mg/dL) and N-acetyl-β-D-glucosaminidase (17.2 U/L) were increased. These results indicated tubulointerstitial damage. Since her baseline creatinine level was not known, in order to explore the cause of acute kidney injury, several tests and examinations were performed. Ultrasound did not reveal any abnormalities in the urinary tract or renal arteries. The levels of the IgG and IgG subclasses, serum, and urine immunofixation electrophoresis were normal. The tests for anti-glomerular basement membrane (GBM) antibodies, and p-ANCA and anti-MPO antibodies were negative. However, the results for c-ANCA and anti-RP3 antibodies were positive. No lesions were found in the sinus or lung by the CT scan. Thus, the diagnosis of granulomatosis with polyangiitis (GPA) was considered. Oral steroids (methylprednisolone 60 mg once daily, decreasing the dose every two weeks by 5 mg) were used. After one month, the serum creatinine and urea nitrogen levels were decreased significantly to 213.4 umol/L and 10.67 mmol/L, respectively. The level of albumin was increased to normal; however, urine total protein and urine α1-microglobulin were still increased. We suppose the loss of kidney function has occurred for some time. And the tubulointerstitial injury was probably due to both hepatorenal syndrome and ANCA-associated small-vessel vasculitis.

Liver transplantation was finally performed in 2024. Methylprednisolone (8 mg once per day), tacrolimus (0.5 mg twice per day), and mycophenolate mofetil (540 mg twice per day) were used for long-term anti-rejection therapy. UDCA was still continued. Until June 2024, the patient was stable. GPA and UC were all in remission.

## 3. Discussion

Extraintestinal manifestations (EIMs) are prevalent in patients with ulcerative colitis (UC). Over a third of patients develop at least one EIM [2]. They most commonly affect musculoskeletal (5.6–30.7%) [7,8], dermatologic (2.6–9.3%) [9,10], ocular (1.7–22.9%) [11,12], and hepatobiliary (0.67–5.10%) [13] systems. Multiple EIMs are not rare (3–7.5%). UC patients who have one EIM seem to be at an increased risk of developing additional EIMs [14,15].

The prevalence of primary biliary cirrhosis (PBC) in UC patients has been reported. M. Koulentaki et al. found that compared to the general population, UC patients seem to have a 30 times higher risk of developing PBC [16]. However, the prevalence of IBD was not increased among PBC patients compared to non-PBC patients [17]. In our database, we identified 4 patients presenting both UC and PBC among 890 UC patients. We calculated the proportion of PBC among UC was 0.45%. Compared to the prevalence number (0.021%, 204.87 cases per million) among the general population in China [18], UC patients seem to have a 21 times higher risk of developing PBC based on our data.

We searched the PubMed database for case reports published in English or with an English abstract up to 31 December 2023 using the keywords “ulcerative colitis” and “primary biliary cholangitis” (or “primary biliary cirrhosis”). There are only 24 cases of UC coexisting with PBC to have been reported [19,20,21,22]. The characteristics of these cases, including our own, are summarized in Table 1.

UC occurs slightly more frequently in men (60%), whereas PBC overwhelmingly affects women (90%). However, the sex ratio differs in patients with UC and PBC (19 women, 9 men). The peak age for UC occurrence is 30–40 years. By contrast, the average age in patients with UC and PBC is higher (46.75 years). Most patients were diagnosed with UC prior to PBC (20/28), while two patients developed these two diseases at the same time, and six patients were detected with UC after PBC. In total, 42.9% of patients of UC with PBC (12 in 28) have more than one manifestation. However, only 3%–7.5% UC patients have at least two EIMs [14,15]. Once UC patients are complicated with PBC, they might have more chances of experiencing more than one EIM. Among all manifestations described in Table 1, psoriasis, peripheral arthritis, ankylosing spondylitis, and PSC are related to UC [5,23], while sclerodactyly, Raynaud’ s phenomenon, SS (including dry mouth), and thyroiditis are connected with PBC [17]. Chronic pancreatitis is considered in association with either UC or PBC [24]. Autoimmune enteropathy (case 24) was believed to be related to a dysregulated immune condition triggered by a microbial shift after colectomy [22]. We reported a very special case who suffered from UC, PBC, SS, TIN, and ILD (case 25). Since our patient was detected with chronic renal insufficiency prior to the continued 5-ASA use, and no evidence was found for drug-induced kidney injury, tubulointerstitial nephritis (TIN) was suspected in connection with SS [25]. ILD is confirmed to be associated with UC, PBC, and SS [26,27]. The association of granulomatosis with polyangiitis (case 28) and UC, PSC, and PBC was difficult to determine. We suppose its association with other diseases to be incidental, although this was a small chance. Other complications listed in Table 1 are not considered to be related to UC or PBC.

**Table 1:** Summary of reported cases of UC associated with PBC.

Case	Sex	Age at UC Diagnosis	UC Prior to PBC	Other Manifestations	Complications	UC Treatment	PBC Treatment
1 [28]	F	65	Yes	Chronic pancreatitis		SASP	
2 [29]	F	47	Yes	Psoriasis, peripheral arthritis	Asthma	SASP, CS	
3 [29]	M	26	Yes			SASP	D-penicillamine
4 [29]	M	44	Yes			SASP, CS	
5 [29]	F	28	Yes			SASP	
6 [30]	F	49	Simultaneously		Chronic myelocytic leukemia, diabetes mellitus	SASP	
7 [31]	M	40	Yes			5-ASA, CS	
8 [32]	F	42	Yes	Chronic pancreatitis, Raynaud’ s phenomenon		5-ASA	UDCA
9 [33]	F	48	Yes		Renal cell carcinoma	5-ASA, CS, AZA	
10 [16]	M	50	Yes	Sclerodactyly, Raynaud’s phenomenon		5-ASA, CS	
11 [16]	F	36	Yes	Dry mouth		SASP	UDCA
12 [34]	F	43	Yes			Proctocolectomy	
13 [35]	M	61	No			SASP, CS	
14 [36]	F	50	No			SASP	UDCA
15 [37]	M	73	Yes		Vitiligo, myasthenia gravis	CS	UDCA
16 [38]	M	64	No		Diabetes mellitus	5-ASA	UDCA
17 [38]	F	40	No	Peripheral arthritis?		5-ASA	UDCA
18 [39]	F	43	No			5-ASA, CS	UDCA
19 [20]	F	50	Yes	Ankylosing spondylitis?	Hypertension, hypercholesterolaemia	5-ASA	UDCA
20 [20]	M	59	Yes	Ankylosing spondylitis	Diabetes mellitus	5-ASA, ADM	UDCA
21 [21]	F	23	Yes			5-ASA	UDCA
22 [21]	F	45	Yes			5-ASA	UDCA
23 [21]	F	47	Yes	Thyroiditis		5-ASA	UDCA
24 [22]	F	49	Yes	Autoimmune enteropathy		CS, proctocolectomy	UDCA
25 (ours)	M	44	Yes	Sjogren’s syndrome, tubulointerstitial nephritis, interstitial lung disease		5-ASA, CS, IFX, UST	UDCA
26 (ours)	F	33	Simultaneously			5-ASA, CS	UDCA, Liver transplantation
27 (ours)	F	63	Yes			5-ASA	UDCA
28 (ours)	F	47	No	PSC, granulomatosis with polyangiitis		VDZ	UDCA, Liver transplantation

Abbreviations: SASP: Sulfasalazine; 5-ASA: 5-Aminosalicylates; CS: Corticosteroids; AZA: Azathioprine; ADM: Adalimumab; IFX: Infliximab; UST: ustekinumab; VDZ: Vedolizumab, UDCA: ursodeoxycholic acid.

For the majority of cases in Table 1, the successful remission induction and maintenance treatment of UC were achieved by using traditional drugs, including SASP/5-ASA, corticosteroids, and immunosuppressants. Only three patients received the treatment of biologic drugs, and one patient underwent surgery. Moreover, UDCA was taken by almost all patients for PBC therapy and the majority of them were under control. Only two patients developed liver cirrhosis and finally underwent liver transplantation. The possible reasons for liver function deterioration may be delayed PBC diagnosis and UDCA usage in case 26, and its accompaniment with PSC in case 27.

The interplay between treatments for PBC and UC should be carefully considered in patient management. UDCA is the primary medication for PBC therapy. Interestingly, it may also have therapeutic potential in IBD due to its immunosuppressant, anti-inflammatory, and cytoprotective actions [40]. However, since UDCA may cause diarrhea, clinicians should take its potential side effects into account in UC assessment. The therapeutic drugs for UC, such as SASP/5-ASA, corticosteroids (CS), immunosuppressants (e.g., azathioprine), and biologic agents, may induce liver injury. Therefore, it is crucial to monitor liver function regularly and compare any changes against the baseline levels in patients undergoing medication therapy.

The reason for developing PBC following UC is not clear. A possible mechanism involves genetic, environmental, and autoimmune components. TNFSF15 has been identified as a susceptibility locus for UC [41]. Interestingly, it was also associated with an increased susceptibility to PBC [42]. Thus, shared genetic factors may contribute to the development of both UC and PBC. Moreover, Mendelian randomization (MR) has been used to analyze the public UC and PBC genome-wide association studies (GWAS) data. The outcomes indicated that genetically predicted UC was significantly associated with an increased risk of PBC. Conversely, genetic susceptibility to PBC might not alter the risk of UC [43]. These results support the clinical observations that UC patients have an increased risk of PBC; however, the prevalence of IBD is not increased among PBC patients.

Gut microbial imbalance is a key factor in the pathogenesis of UC. Increased harmful bacteria (such as *Escherichia coli*) or reduced beneficial bacteria (such as *Lactobacillus* and *Bifidobacterium*) can compromise the integrity of the intestinal barrier, allowing pathogens to penetrate the intestinal wall and trigger immune responses [44]. Importantly, there is an increasing emphasis on the effect of gut microbiota in PBC via the gut–liver axis. For example, Yao Yang et al. demonstrated that an exposure to *Escherichia coli* elicits specific antibodies to ePDC-E2, resulting in the development of PBC [45]. Thus, environmental factors such as dysbiosis and autoimmune components may be involved in the pathogenesis of both UC and PBC.

## 4. Conclusions

UC with PBC is uncommon. However, compared to the general population, UC patients seem to have a higher risk of developing PBC. Patients usually develop UC prior to PBC. Once UC patients are complicated with PBC, they might experience more than one extraintestinal manifestation (EIM). Shared susceptibility genes, certain bacterial infections, and common immune-mediated mechanisms may be involved in the pathogenesis of UC in patients with PBC.

## Data Availability

The data generated in the present study are included in the figures of this article. The data can be obtained from the authors upon making a reasonable request.
